# Interaction between cytochrome P450 2A6 and Catechol-*O*-Methyltransferase genes and their association with smoking risk in young men

**DOI:** 10.1186/s12993-017-0127-2

**Published:** 2017-05-04

**Authors:** Wei-Chih Ou, Yi-Chin Huang, Chih-Ling Huang, Min-Hsuan Lin, Yi-Chun Chen, Yi-Ju Chen, Chen-Nu Liu, Mei-Chih Chen, Ching-Shan Huang, Pei-Lain Chen

**Affiliations:** 1 Department of Medical Laboratory Science and Biotechnology, Central Taiwan University of Science and Technology, No. 666 Buzih Road, Beitun District, Taichung City, 40601 Taiwan; 2 Department of Nursing, Chang Jung Christian University, Tainan, Taiwan; 3 Administration Center for Research and Education, Changhua Christian Hospital, Changhua, Taiwan; 4 Company Limited of Ditech Enterprise, Taipei, Taiwan; 5 Division of Infectious Diseases, Jen-Ai Hospital, Taichung, Taiwan

**Keywords:** Catechol-*O*-methyltransferase, Cotinine, Cytochrome P450 2A6, Nicotine, Smoking status

## Abstract

**Background:**

Although some effects of gene–gene interactions on nicotine–dopamine metabolism for smoking behavior have been reported, polymorphisms of cytochrome P450 (CYP) 2A6 and catechol-*O*-methyltransferase (COMT) have not been studied together to determine their effects on smokers. The aim of this study was to investigate the effects of the interaction between the *CYP* 2A6 and *COMT* genes on smoking behavior in young Taiwanese men.

**Results:**

A self-report questionnaire regarding smoking status was administered to 500 young men. Polymorphisms of the *CYP 2A6* and *COMT* genes as well as urinary nicotine and urinary cotinine levels were determined. The odds ratio for starting smoking was significantly lower in subjects carrying a *CYP2A6* low activity/variant *COMT* rs4680 genotype than in those possessing a *CYP2A6* wild-type/variant *COMT* rs4680 genotype (0.44, 95% confidence interval = 0.19–0.98, P = 0.043). Comparisons of Fagerstrom Test for Nicotine Dependence (FTND), Physiological Cigarette Dependence Scale (PCDS), and Cigarette Withdrawal symptoms (CWS-21) among the smokers with different *CYP2A6/COMT* polymorphisms were not significantly different. The adjusted urinary nicotine concentrations were not significantly different between the two groups carrying different genotypes. The adjusted urinary cotinine level was significantly different between the *COMT* rs4680 wild-type group and *COMT* rs4680 variant group [92.46 ng/μL vs. 118.24 ng/μL (median value), P = 0.041] and between the *COMT* rs4680 wild-type/*COMT* rs165599 variant group and *COMT* rs4680 variant/*COMT* rs165599 variant group (97.10 ng/μL vs. 122.18 ng/μL, P = 0.022).

**Conclusions:**

These findings suggest that a single nucleotide polymorphism (rs4680) of the *COMT* gene and the interaction between the *CYP* 2A6 and *COMT* genes affect smoking status in young Taiwanese men.

**Electronic supplementary material:**

The online version of this article (doi:10.1186/s12993-017-0127-2) contains supplementary material, which is available to authorized users.

## Background

Tobacco smoking is a multi-factorial behavior with both genetic and environmental determinants [1]. Genetic factors have a greater influence on smoking cessation than do environmental factors [2]. Genetic factors are responsible for 30–50% of the variance in the risk of withdrawal symptoms, 40–75% of the variation in smoking initiation, 50% of the variance in cessation success, and 70–80% of the variation in smoking maintenance [1]. Genetic risk information enhances the motivation for smoking cessation [3]. Therefore, assessment of genetic background could be a promising tool to understand smoking risk and to guide the selection of the most effective cessation treatment for an individual smoker.

Nicotine is the major psychoactive ingredient in tobacco, and it modulates dopamine activity in the midbrain, which contributes to the development and maintenance of rewarding behaviors such as smoking [4]. Smokers modulate their smoking to maintain brain nicotine levels within a certain concentration range, and factors that alter nicotine clearance affect smoking behavior [4]. Individuals who eliminate nicotine rapidly are less likely to achieve low craving scores even after smoking freely [4]. Consequently, genetic polymorphisms in both nicotine metabolism and dopamine catabolism genes influence smoking status, interact with each other to result in risk modulation, and affect smoking cessation therapies.

The cytochrome P450 (*CYP*) 2A6 gene, located on chromosome 19q12-q13.2, consists of nine exons. It is involved in producing a 494-amino-acid protein that oxidizes coumarin, nicotine, and tobacco-specific nitrosamines [5]. *CYP2A6* is the primary human enzyme involved in nicotine metabolism [4]. *CYP2A6* catalyzes the C-oxidation of nicotine to the inactive metabolite cotinine and the subsequent conversion of cotinine into trans-3′-hydroxycotinine [4]. *CYP2A6* is the most studied enzyme involved in both adult and adolescent smokers [6]. The results of studies among Taiwanese individuals indicate that the variant status of *CYP2A6* is different from that among other ethnic groups [7]. Therefore, we hypothesized that, for Taiwanese individuals, polymorphisms in the *CYP2A6* gene that affect smoking status could be different from those in other ethnic groups.

The catechol-*O*-methyltransferase (*COMT)* gene is located on chromosome 22q11.21, has eight exons, and produces a 271-amino-acid protein that metabolizes catecholamines [8]. Low enzyme activity of the Met allele at codon 108/158 (in the rs4680 polymorphism) of the *COMT* gene, which encodes a key enzyme involved in the metabolic inactivation of dopamine, has been associated with nicotine dependence [9]. Another polymorphism of *COMT*, rs165599, has been related to the response to bupropion therapy for smoking cessation [10]. However, those associations have been inconsistent among ethnic groups [10, 11]. Thus, it seems necessary to perform a genetic study of smoking status for each ethnicity.

Smoking addiction is currently a significant social problem in Taiwan [12]. Nonetheless, very few genetic investigations of nicotine–dopamine metabolism and smoking status among Taiwanese individuals have been performed. Recently, for Taiwanese smokers in a group of methadone maintenance patients, polymorphisms of the μ-opioid receptor gene were associated with the plasma concentration of cotinine [13]. Very recently, our team reported that the interaction of the dopamine D2 receptor (*DRD2*) TaqIB and monoamine oxidase A (*MAOA*) affected smoking intensity in young men [14]. Those findings encouraged us to perform more genetic studies of Taiwanese smokers. In relation to nicotine–dopamine metabolism, smoking status has been reported to correlate better with some gene–gene interactions than with a single gene only. These interactions include *COMT* and *MAOA* [15, 16], *MAOA* and *CYP2A6* [17], *CYP2A6* and the nicotine acetylcholine receptor gene [18, 19], and *CYP 2A6* and *DRD2* TaqIA [20]. However, the effects of *CYP2A6* and *COMT* together have not been explored, and we hypothesized that a single polymorphism as well as an interaction of the two genes could be involved in smoking status.

In women not using oral contraceptives, nicotine and cotinine clearance is 13 and 24% higher, respectively, than in men [6]. Sex differences exist for cravings, affect, and preference for immediate smoking after cue exposure [21]. Because Taiwanese men smoke significantly more cigarettes than Taiwanese women [12, 22], only men were invited to participate in our study. In this study, the polymorphisms CYP2A6*1A (wild-type), CYP 2A6*1B (polymorphism of faster nicotine clearance) [23], CYP2A6*4C (the most studied polymorphism of decreased nicotine metabolism) [24], *COMT* Val/Met (rs4680) [9], and *COMT* rs165599 [10] were determined. Our objectives were to investigate the effects of the interaction between the *CYP 2A6* and *COMT* genes and their association with smoking risk in young Taiwanese men.

## Methods

### Participants and procedures

This cross-sectional study was advertised to all students at both Chang Jung Christian University and Central Taiwan University of Science and Technology. The volunteers contacted the authors of the study, and convenience samples were then screened for eligibility at the health centers of the two universities. All study subjects provided written, informed consent at the beginning of study, and the study was approved by the review board of Chang Jung Christian University (CJCU-99004) and Central Taiwan University of Science and Technology (CTUST-99016). The study was conducted in accordance with Good Clinical Practice procedures and the Declaration of Helsinki.

We administered a self-report questionnaire to all of the study subjects [25–27]. The questionnaire included demographic data, smoking background and status. In addition, the questionnaire included the fagerstrom test for nicotine dependence (six-item FTND) [25], the 15-item short form of the Physiological Cigarette Dependence Scale (PCDS) derived from the 30-item PCDS [27], and the Withdrawal Symptoms Scale (Cigarette Withdrawal Scale, CWS-21) [26, 27]. These questionnaires used biomarkers (nicotine and cotinine concentrations) as validation, with no over- or under-exaggeration. Never smokers were defined as persons who had never smoked in their lifetime. Current smoking was defined as ever smoking cigarettes on 1 or more days of the past 30 days. Ever smokers were defined as persons who smoked at one time, had quit, and were not currently smoking. The current smokers were divided into two groups according to their intensity of cigarette smoking: light smokers and heavy smokers, depending on number of cigarettes per day lower than or equal to (or higher than) the average value of all the smokers, respectively. The exclusion criteria were (1) a history of diagnosed mental health disease or cancer, (2) alcoholism or drug abuse, (3) severe communication problems, or (4) terminal illness. When the survey was completed, blood and urine samples were obtained at a university center by trained research assistants (licensed nurses or medical technologists).

### Determination of CYP2A6 and COMT polymorphisms

In this study, to determine the genotypes of the variants of interest, total genomic DNA was isolated from blood cells using a blood DNA isolation kit (Favorgen, Ping-Tung, Taiwan). Five milliliters of whole blood with EDTA as an anticoagulant were required. Approximately 40 alleles of the *CYP2A6* gene have been identified [28]. However, only the wild-type (CYP2A6*1A) and two highly prevalent (>10%) variants of CYP2A6*1B (approximately 45%) [29] and CYP2A6*4C (approximately 15%) [30], which have been observed in Chinese individuals, were determined in this project.

The polymorphisms of *CYP2A6* were identified using polymerase chain reaction-restriction fragment length polymorphism (PCR–RFLP) [31]. We designed the forward primer 5′-CACCGAAGTGTTCCCTATGCTG-3′ and reverse primer 5′-TGTAAAATGGGCATGAACGCCC-3′ according to the GenBank accession system. PCR was performed with a thermal cycler (Bio-Rad, Carlsbad, CA, USA). The PCR conditions were as follows: first cycle, denaturation at 94 °C for 3 min; cycles 2–31, denaturation at 94 °C for 1 min, annealing at 55 °C for 1 min, and elongation at 72 °C for 2 min, with a final extension for 7 min at 72 °C. We detected a 1332-base pair (bp) fragment on a 1% agarose gel after electrophoresis at 100 V for 60 min. Using *BstU*-*I* as the restriction enzyme, one (1332-bp) and two (291 and 1041-bp) fragments were obtained for CYP2A6*1A and *CYP2A*6*non-1A, respectively. Using the restriction enzyme *Bsu36*-*I*, three fragments (104, 437, and 792-bp) were observed for CYP2A6*1A or CYP2A6*1B. Four fragments (64, 104, 437, and 728-bp) were found for CYP2A6*4C. Positive controls were run for each of the genotyping assays. The DNAs of CYP2A6*1A/*1A, CYP2A6*1A/*1B, CYP2A6*1B/*1B, CYP2A6*1A/*4C, and CYP2A6*1B/*4C, which have been found in Taiwanese and Chinese populations, were identified by DNA sequencing [29, 30]. Additional file 1: Table S1 lists the result.

For *COMT* rs4680, the forward primer 5′-CTGTGGCTACTCAGCTGTG-3′ and reverse primer 5′-CCTTTTTCCAGGTCTGACAA-3′ were used to amplify a 169-bp fragment [10]. Using *N1a III* as the restriction enzyme, three (114, 32, and 23-bp), four (96, 32, 23, and 18-bp), and five (114, 96, 32, 23, and 18-bp) fragments were obtained for the G/G, A/A, and G/A genotypes, respectively [10]. For COMT rs165599, the forward primer 5′-CATTCAAAGCTCCCCTTGAC-3′ and reverse primer 5′-GGGAGTAGG-GAAGGAGATGC-3′ were utilized to amplify a 301-bp fragment [32]. Using *Msp I* as the restriction enzyme, one (301-bp), two (166 and 135-bp), and three (301, 166, and 135-bp) fragments were obtained from the A/A, G/G, and A/G genotypes, respectively [32].

### Determination of urinary nicotine and cotinine

To evaluate the effects of genetic polymorphisms on the metabolism of nicotine and cotinine, urinary nicotine and cotinine levels of smokers were measured. Gas chromatography–mass spectrometry (GC/MS) was performed as previously described [33], with the following difference: GC–MS analyses were performed on a ThermoElectron DSQII quadrupole mass spectrometer connected directly to a ThermoElectron focus gas chromatograph and an autosampler AS 3000 (Thermo Electron Corporation, Dreieich, Germany). All urine samples were stored at −20 °C before analysis. All of the analyses were performed in duplicate and repeated if values differed by >10%. Tobacco cigarettes currently smoked in Taiwan contain 0.57–0.64 mg of nicotine per cigarette on average [12]. The nicotine and cotinine concentrations in the urine of each subject were divided by the number of daily cigarettes smoked and defined as the adjusted nicotine and adjusted cotinine concentration.

### The effects of gene polymorphism interactions on smoking behaviors

The *CYP2A6* genotypes consisted of wild-type (*1A/*1A), high -activity (*1A/*1B and *1B/*1B), and low-activity (*1A/*4C, *1B/*4C, and *4C/*4C) genotypes. The *COMT* rs4680 genotypes consisted of wild-type (G/G) and variant (G/A and A/A). The *COMT* rs165599 genotypes consisted of wild-type (A/A) and variant (A/G and G/G) genotypes. To assess the interaction between the effects of *CYP2A*6 and *COMT* gene polymorphisms on smoking behaviors, multiple models were used to analyze the *CYP2A6*, *COMT* rs4680, and *COMT* rs165599 data: (1) single gene (model 1: *CYP2A*6) or single SNP (model 2: *COMT* rs4680, *COMT* rs165599, respectively); (2) 2 SNPs (model 3: *COMT* rs4680 and *COMT* rs165599); and (3) multiple genes (models 4–7). We evaluated the effect of the interactions between different gene combinations on smoking status (Table 2), smoking intensity (Additional file 2: Table S2), nicotine dependence (FTND), physiological cigarette dependence (PCDS), nicotine toxicity and withdrawal symptoms (CWS-21) (Additional file 3: Table S3), and urine nicotine/cotinine concentration (Table 3) in young men.

### Statistical analysis

To evaluate the effect of the genetic variants on smoking status and smoking intensity, this study assigned an odds ratio (OR) as 1 to subjects carrying the wild-type. The Mantel–Haenszel Chi square test was utilized to calculate the ORs and their 95% confidence intervals (CIs) (Table 2; Additional file 2: Table S2). To compare the significance of FTND, PCDS, and CWS-21 and to compare the urinary nicotine and cotinine clearance among subjects carrying different *CYP2A6* and *COMT* polymorphisms, Student’s *t* test or analysis of variance (ANOVA), as appropriate, was applied to compare quantitative data. A P value of <0.05 was defined as statistically significant for each analysis (Additional file 3: Table S3). When the result of ANOVA was statistically significant, multiple comparisons were followed by application of Scheffé’s post hoc test. However, if the data in any group did not fit a normal distribution, the Mann–Whitney U test with Bonferroni adjustment was utilized to compare the data between the two groups (Table 3). All data were analyzed using SPSS (version 18.0 software for Windows, SPSS Inc., Chicago, IL).

## Results

We recruited a total of 500 men aged 20–25 years. Analysis of the questionnaires revealed that there were 219 never smokers, 261 current smokers, and 20 ever smokers. Due to the small sample size (moderate power analysis in general, α = 0.05 and 80% power requires 200 samples), the 20 ever smokers were excluded from further study. Mean age did not differ between the 261 current smokers and the 219 never smokers (22.6 ± 1.69 vs. 22.4 ± 1.42, *t* = 1.371, P = 0.176, data not shown).

The distributions of *CYP2A6* and *COMT* polymorphisms are shown in Table 1. Using the frequencies of CYP2A6*1A/*1A, CYP2A6*1A/*1B, and CYP2A6*1B/*1B as examples, the distributions of *CYP2A6* genotypes agreed with Hardy–Weinberg equilibrium: P = 0.713 and 0.332 for current smokers and never smokers, respectively. The distribution of *COMT* rs165599 genotypes also agreed with Hardy–Weinberg equilibrium: P = 0.966 and 0.668 for current smokers and never smokers, respectively. The distribution of *COMT* rs4680 genotypes did not agree with Hardy–Weinberg equilibrium: P = 0.003 and 0.012 for current smokers and never smokers, respectively.

**Table 1:** Distribution of CYP2A6 and COMT polymorphisms

Genotypes	Current smokers [n = 261 (n%)]	Hardy–Weinberg equilibrium	Never smokers [n = 219 (n%)]	Hardy–Weinberg equilibrium
CYP2A6		P^a^ = 0.713		P^a^ = 0.332
1A/1A	63 (24.1)		46 (21.0)	
1A/1B	100 (38.3)		88 (40.2)	
1B/1B	44 (16.9)		31 (14.2)	
1A/4C	24 (9.2)		25 (11.4)	
4C/4C	4 (1.5)		3 (1.37)	
1B/4C	26 (10.0)		26 (11.9)	
COMT rs4680		P^b^ = 0.003		P^b^ = 0.012
G/G	147 (56.3)		113 (51.6)	
G/A	73 (28.0)		69 (31.5)	
A/A	41 (15.7)		37 (16.9)	
COMTrs165599		P^c^ = 0.966		P^c^ = 0.668
A/A	61 (23.4)		45 (20.6)	
A/G	130 (49.8)		112 (51.1)	
G/G	70 (26.8)		62 (28.3)	

^a^For CYP2A6 1A/1A, 1A/1B, 1B/1B

^b^For COMT rs4680

^c^For COMT rs165599

As shown in Table 2, after using never smokers as the reference group, the OR was significantly lower in the subjects carrying the genotype of *CYP2A6* low activity/variant *COMT* rs4680 than in those possessing the genotype of *CYP2A6* wild-type/variant *COMT* rs4680 (0.44, 95% CI 0.19–0.98, P = 0.043). The other 14 ORs were not statistically significant (P = 0.078–0.988).

**Table 2:** Odds ratios for the effects of *CYP2A6* and *COMT* polymorphisms on smoking status

Genotypes interaction	Current smokers N = 261	Never smokers N = 219	OR (95% CI)	P^a^
Model 1				
CYP2A6				
Wild type	63	46	1.0	
High activity	144	119	0.88 (0.56–1.39)	0.591
Low activity	54	54	0.73 (0.43–1.25)	0.249
Model 2				
COMT				
COMT rs4680				
Wild type	147	113	1.0	
Variant	114	106	0.83 (0.58–1.19)	0.301
COMT rs165599				
Wild type	61	45	1.0	
Variant	200	174	0.85 (0.55–1.31)	0.458
Model 3				
COMT rs4680/COMT rs165599				
Wild type/wild type	27	13	1.0	
Wild type/variant	120	100	0.58 (0.28–1.18)	0.128
Variant/wild type	34	32	0.51 (0.23–1.16)	0.107
Variant/variant	80	74	0.52 (0.25–1.08)	0.078
Model 4				
COMT rs4680 wild type				
CYP2A6 wild type	35	25	1.0	
CYP2A6 high activity	76	65	0.84 (0.45–1.54)	0.563
CYP2A6 low activity	36	23	1.12 (0.54–2.33)	0.765
Model 5				
COMT rs4680 variant				
CYP2A6 wild type	28	21	1.0	
CYP2A6 high activity	68	54	0.94 (0.48–1.84)	0.867
CYP2A6 low activity	18	31	0.44 (0.19–0.98)	0.043
Model 6				
COMT rs165599 wild type				
CYP2A6 wild type	15	8	1.0	
CYP2A6 high activity	33	30	0.59 (0.22–1.58)	0.289
CYP2A6 low activity	13	7	0.99 (0.28–3.48)	0.988
Model 7				
COMT rs165599 variant				
CYP2A6 wild type	48	38	1.0	
CYP2A6 high activity	111	89	0.99 (0.59–1.64)	0.961
CYP2A6 low activity	41	47	0.69 (0.38–1.26)	0.224

^a^Mantel–Haenszel Chi square test

The genotypes of (1) CYP2A6: wild-type (*1A/*1A), high activity (*1A/*1B and *1B/*1B), low activity (*1A/*4C, *1B/*4C, and *4C/*4C); (2) COMT rs4680: wild-type (G/G), variant (G/A and A/A); and (3) COMT rs165599: wild-type (A/A), variant (A/G and G/G)

The average number of cigarettes per day among the 261 current smokers was 10. There were 127 heavy smokers and 131 light smokers (data for three smokers were missing). With light smokers as the reference group, 15 ORs for heavy smoking were not statistically significant (P = 0.061–0.112, data in Additional file 2: Table S2).

For the 261 current smokers, FTND, PCDS, and CWS-21 were analyzed for 181, 227, and 210 subjects, respectively, because of missing data. Each of the eight comparisons for FTND, PCDS, and CWS-21 among the groups with different *CYP2A6/COMT* polymorphisms was not significantly different (P = 0.224–0.911, 0.054–0.700, and 0.075–0.836, respectively) (data in Additional file 3: Table S3).

Of the 261 current smokers, 122 subjects provided urine samples for the determination of nicotine and cotinine levels by GC/MS. The values for adjusted nicotine concentration and adjusted cotinine concentration were not normally distributed in either genotype group. The SD was too high: near the mean value or even greater than the mean value. Therefore, the Mann–Whitney U test or Kruskal–Wallis test with Bonferroni adjustment was used for comparison of the values of adjusted nicotine concentration and adjusted cotinine concentration between groups carrying different genotypes. As shown in Table 3, the adjusted urinary nicotine concentration did not differ significantly in any of the comparisons. The median adjusted urinary cotinine concentration differed between the *COMT* rs4680 wild-type group and the *COMT* rs4680 variant group (92.46 ng/μL vs. 118.24 ng/μL, P = 0.041) and between the *COMT* rs4680 wild-type/*COMT* rs165599 variant group and the *COMT* rs4680 variant/*COMT* rs165599 variant group (97.10 ng/μL vs. 122.18 ng/μL, P = 0.022). The other comparisons were not statistically significant.

**Table 3:** Comparisons of adjusted urinary nicotine concentration and adjusted urinary cotinine concentration among subjects (N = 122) carrying different CYP2A6 and COMT polymorphisms

	N	Adjusted urinary nicotine, ng/μL median (min–max)	Adjusted urinary cotinine, ng/μL median (min–max)
Model 1			
CYP2A6			
Wild type	17	86.23 (6.14–296.36)	120.90 (20.39–485.32)
High activity	76	70.45 (6.28–1117.87)	111.08 (8.34–822.11)
Low activity	29	61.59 (15.28–456.87)	75.87 (10.87–266.44)
P^a^		NS	NS
Model 2			
COMT			
COMT rs4680			
Wild type	66	63.86 (6.28–646.38)	92.46 (8.34–506.97)
Variant	56	67.87 (6.14–1117.87)	118.24 (13.59–822.11)
P^a^		NS	0.041
COMT rs165599			
Wild type	27	68.10 (12.42–212.74)	89.42 (23.06–218.62)
Variant	95	64.11 (6.14–1117.87)	107.24 (8.34–822.11)
P^a^		NS	NS
Model 3			
COMT rs4680/COMT rs165599			
Wild type/wild type	13	89.29 (12.42–212.74)	79.37 (23.06–218.62)
Wild type/variant	53	63.61 (6.28–646.38)	97.10 (8.34–506.97)*
Variant/wild type	14	67.87 (15.28–177.61)	95.86 (32.18–202.0)
Variant/variant	42	69.46 (6.14–1117.87)	122.18 (13.59–822.11)*
P^a^		NS	
Model 4			
COMT rs4680 wild type			
CYP2A6 wild type	9	95.04 (25.04–296.36)	105.70 (53.48–485.32)
CYP2A6 high activity	39	72.99 (6.28–646.38)	97.45 (8.34–506.97)
CYP2A6 low activity	18	42.54 (19.38–371.29)	60.20 (10.87–266.44)
P^a^		NS	NS
Model 5			
COMT rs4680 variant			
CYP2A6 wild type	8	74.91 (6.14–217.41)	141.53 (20.39–228.68)
CYP2A6 high activity	37	68.10 (17.68–1117.87)	115.78 (13.59–822.11)
CYP2A6 low activity	11	66.13 (15.28–456.87)	161.65 (39.19–224.53)
P^a^		NS	NS
Model 6			
COMT rs165599 wild type			
CYP2A6 wild type	3	95.04 (62.36–188.57)	102.30 (79.37–120.90)
CYP2A6 high activity	17	67.64 (12.42–212.74)	89.42 (32.18–218.62)
CYP2A6 low activity	7	78.05 (15.28–177.61)	63.55 (23.06–202.00)
P^a^		NS	NS
Model 7			
COMT rs165599 variant			
CYP2A6 wild type	14	74.92 (6.14–296.36)	141.53 (20.39–485.32)
CYP2A6 high activity	59	72.99 (6.28–1117.87)	111.09 (8.34–822.11)
CYP2A6 low activity	22	59.62 (19.38–456.87)	81.66 (10.87–266.44)
P^a^		NS	NS

*NS* not statistically significant

^**a**^Mann–Whitney U test or Kruskal–Wallis test with Bonferroni adjustment

* Significantly different from the (COMT rs4680 wild-type/COMT rs165599 variant) group versus the (COMT rs4680 variant/COMT rs165599 variant) group by Scheffe’s Post hoc test, P value = 0.022

## Discussion

All subjects in this study were young adult university students. Alcohol and drug abusers were excluded. Therefore, the effects of age, education status and alcohol and drug interactions on smoking were absent.

Our results show that the distribution of *COMT* rs4680 does not agree with Hardy–Weinberg equilibrium, consistent with reports that Asiatic individuals have Hardy–Weinberg disequilibrium of the *COMT* rs4680 polymorphism [8]. The main finding of this study is that among adult males with variant *COMT* rs4680, the subjects carrying the low-activity genotype of *CYP2A6* have a 0.44-fold lower risk of starting smoking than those possessing the wild-type genotype of *CYP2A6*. In other words, the OR is 2.27-fold higher in subjects carrying the *CYP2A6* wild-type/variant *COMT* rs4680 genotype than in those possessing the *CYP2A6* low activity/variant *COMT* rs4680 genotype. The *CYP2A6* and *COMT* genes were reported to be associated with smoking status. CYP2A6*4C is a whole-deletion type, decreased nicotine metabolism polymorphism [34]. In Japanese adults and young Japanese students, the frequency of the CYP2A6*4C gene was significantly higher among non-smokers than smokers [34, 35]. Among Chinese males, participants with the CYP2A6*4C genotype had a lower risk of smoking initiation and smoking persistence than those with the CYP2A6*1/CYP2A6*1 genotype [17]. For people living in southern China, reduced metabolic function of *CYP2A6* in smokers appears to be associated with fewer cigarettes smoked, later initiation of smoking regularly, shorter smoking duration, and lower likelihood of smoking cessation [36]. For Caucasian individuals, the *CYP2A6* slow inactivator genotype increased the risk of nicotine dependence when smoking was initiated during adolescence. However, it reduced the risk of smoking initiation, lowered cigarette consumption, and decreased the duration of smoking among adult dependent smokers [37]. The continued effect of slow metabolism on reducing cigarette consumption, throughout the smoking history of people with *CYP2A6* slow inactivators, may affect tolerance and withdrawal mechanisms among these individuals [37]. Smokers with *CYP2A6* slow inactivators smoke fewer cigarettes and tend to be less dependent on nicotine than smokers with normal activity alleles [38]. With respect to smoking initiation, adolescents with slower activity alleles may progress to nicotine dependence more slowly than normal metabolizers [38]. Very recently, researchers reported that *CYP2A6* slow metabolism was associated with increased adolescent smoking cessation in Caucasian individuals [39]. The ORs for current smoking are reportedly higher in *COMT* rs4680 G/G (the high activity allele) carriers than in those possessing the *COMT* rs4680 variant among healthy Caucasian men of Croatian origin [40], Americans of European ancestry [41], and Japanese men [15]. On the other hand, the *COMT* rs4680 variation (G/A or A/A, the low-activity alleles) was associated with nicotine dependence in men and women of African-American and European-American descent [9], smoking severity among Chinese male smokers [42], heaviness of smoking in Caucasian pregnant women [43], and susceptibility to cigarette smoking among Thai males [11]. Our results demonstrate for the first time that the combination of the low-activity *CYP2A6* genotype and low-activity *COMT* genotype is associated with the risk of starting smoking.

We also found that the adjusted urinary cotinine concentration was higher in subjects with low-activity *COMT* genes than in those with high-activity *COMT* genes. Additionally, it was higher in subjects with the *COMT* rs4680 variant/*COMT* rs165599 variant than in those with the *COMT* rs4680 wild-type/*COMT* rs165599 variant. The urinary cotinine concentration is a reliable easy-to-use marker for plasma levels of cotinine and the sum of nicotine metabolites in smokers [44]. Therefore, our findings indicate that cotinine and the sum of nicotine metabolites are metabolized more slowly in subjects carrying the low-activity genotype than in those possessing the high-activity genotype. However, such a difference did not affect FTND, PCDS, or CWS-21 scores, and it was not related to the risk of heavier smoking. The smoking intensity of the university students was lower (average cigarettes/day = 10 and average FTND score = 3.7) than that of Taiwanese adult smokers, e.g., average cigarettes/day = 24 and average FTND score = 7.1 for Chinese adults [42]. This finding may be the reason, at least in part, that the low activity genotypes of *CYP2A6* and *COMT* were associated with high nicotine dependence scores and heavier smoking, as we reported in this study. For Taiwanese university students, the primary reason for the first contact with smoking was curiosity. Anxiety, avoiding stress, and the difficulties of smoking cessation explained continuing smoking behavior among university students [45]. The environmental factors may be more predominant than certain genetic factors (e.g., polymorphisms of *CYP2A6* and *COMT* genes) among Taiwanese smokers in the university setting.

There are several limitations to our study. The percentage of female smokers in the Taiwanese population is low, only 4.4%, and they are difficult to recruit as participants. In our report, study participation was restricted to young male Taiwanese smokers. Although the total number of study subjects was relatively large, the numbers of individuals with some of the genotypes were too small to reach statistically significant power. Therefore, further studies with a larger sample size and that include female smokers are needed.

## Conclusions

A single nucleotide polymorphism (rs4680) in the *COMT* gene and the interaction between the *CYP2A6* and *COMT* genes affect smoking status in young Taiwanese men. Effective smoking prevention and cessation intervention programs are required to reduce smoking among university students [46]. We found that the interaction of the low-activity *CYP2A6* genotype and low-activity *COMT* genotype is associated with the risk of starting smoking. In addition, interaction of the *DRD2* TaqIB and *MAOA* genes also affects smoking intensity in Taiwanese young men [14]. This knowledge is useful for developing an approach to reducing smoking among Taiwanese university students. A clearer understanding of the relative roles of genetic and non-genetic factors in the initiation of smoking could have implications for the design of smoking prevention programs [47].

## Additional files

**Additional file 1: Table S1.** Results of PCR–RFLP fragments for CYP2A6.

**Additional file 2: Table S2.** Odds ratios for the effect of CYP2A6 and COMT.

**Additional file 3: Table S3.** Comparisons of FTND, PCDS and CWS-21 among subjects carrying different CYP2A6 and COMT polymorphisms.

## Abbreviations

CYP2A6: cytochrome P450 2A6
COMT: catechol-*O*-methyltransferase
FTND: Fagerstrom Test for Nicotine Dependence
PCDS: Physiological Cigarette Dependence Scale
CWS: Cigarette Withdrawal Symptoms Scale
