# Supplementary material for: Interaction between cytochrome P450 2A6 and Catechol-O-Methyltransferase genes and their association with smoking risk in young men
Source: Behav Brain Funct. 2017 May 4;13:8. doi: 10.1186/s12993-017-0127-2 (PMC5418756; doi:10.1186/s12993-017-0127-2)
Supplement: Supplementary file 1 — Additional file 1: Table S1. Results of PCR–RFLP fragments for CYP2A6. [file 12993_2017_127_MOESM1_ESM.docx]

**Additional file 1: Table S1** Results of PCR-RFLP fragments for CYP2A6 polymorphisms

|  | **BstU-I** | **Bsu36-I** |
| --- | --- | --- |
| CYP2A6*1A/*1A | 1332-bp | 104, 437 and 792-bp |
| CYP2A6*1A/*1B | 291, 1041 and 1332-bp | 104, 437 and 792-bp |
| CYP2A6*1B/*1B | 291 and 1041-bp | 104, 437 and 792-bp |
| CYP2A6*1A/*4C | 291, 1041 and 1332-bp | 64, 104, 437, 728 and 792-bp |
| CYP2A6*4C/*4C | 291 and 1041-bp | 64, 104, 437 and 728-bp |
| CYP2A6*1B/*4C | 291 and 1041-bp | 64, 104, 437, 728 and 792-bp |
