# Supplementary material for: Interaction between cytochrome P450 2A6 and Catechol-O-Methyltransferase genes and their association with smoking risk in young men
Source: Behav Brain Funct. 2017 May 4;13:8. doi: 10.1186/s12993-017-0127-2 (PMC5418756; doi:10.1186/s12993-017-0127-2)
Supplement: Supplementary file 2 — Additional file 2: Table S2. Odds ratios for the effect of CYP2A6 and COMT. [file 12993_2017_127_MOESM2_ESM.docx]

**Additional file 2: Table S2** Odds ratios for the effect of CYP2A6 and COMT polymorphisms on heavy versus light smoking

|  | **Heavy smokers**  **N = 164** | **Light smokers**  **N = 94** | **OR**  **(95% CI)** | **P^a^** |
| --- | --- | --- | --- | --- |
| **Model 1** |  |  |  |  |
| **CYP2A6** |  |  |  |  |
| Wild type | 41 | 19 | 1.0 |  |
| High activity | 85 | 59 | 0.69 (0.38–1.26) | 0.224 |
| Low activity | 38 | 16 | 1.10 (0.50–2.45) | 0.814 |
| **Model 2** |  |  |  |  |
| **COMT** |  |  |  |  |
| COMT rs4680 |  |  |  |  |
| Wild type | 95 | 51 | 1.0 |  |
| Variant | 69 | 43 | 0.86 (0.52–1.44) | 0.567 |
| COMT rs165599 |  |  |  |  |
| Wild type | 42 | 16 | 1.0 |  |
| Variant | 122 | 78 | 0.60 (0.31–1.13) | 0.112 |
| **Model 3** |  |  |  |  |
| COMT rs4680/COMT rs165599 |  |  |  |  |
| Wild type/wild type | 18 | 8 | 1.0 |  |
| Wild type/variant | 77 | 43 | 0.80 (0.32–1.98) | 0.623 |
| Variant/wild type | 24 | 8 | 1.33 (0.42–4.23) | 0.625 |
| Variant/variant | 45 | 35 | 0.57 (0.22–1.47) | 0.242 |
| **Model 4** |  |  |  |  |
| **COMT rs4680 wild type** |  |  |  |  |
| CYP2A6 wild type | 26 | 8 | 1.0 |  |
| CYP2A6 high activity | 44 | 32 | 0.42 (0.17–1.06) | 0.061 |
| CYP2A6 low activity | 25 | 11 | 0.70 (0.24–2.03) | 0.509 |
| **Model 5** |  |  |  |  |
| **COMT rs4680 variant** |  |  |  |  |
| CYP2A6 wild type | 15 | 11 | 1.0 |  |
| CYP2A6 high activity | 41 | 27 | 1.11 (0.45–2.79) | 0.818 |
| CYP2A6 low activity | 13 | 5 | 1.91 (0.52–6.94) | 0.325 |
| **Model 6** |  |  |  |  |
| **COMT rs165599 wild type** |  |  |  |  |
| CYP2A6 wild type | 9 | 3 | 1.0 |  |
| CYP2A6 high activity | 23 | 10 | 0.77 (0.17–3.45) | 0.729 |
| CYP2A6 low activity | 10 | 3 | 1.11 (0.18–6.97) | 0.910 |
| **Model 7** |  |  |  |  |
| **COMT rs165599 variant** |  |  |  |  |
| CYP2A6 wild type | 32 | 16 | 1.0 |  |
| CYP2A6 high activity | 62 | 49 | 0.63 (0.31–1.28) | 0.203 |
| CYP2A6 low activity | 28 | 13 | 1.08 (0.44–2.62) | 0.870 |

^a^Mantel-Haenszel chi-square test.
