# Supplementary material for: Interaction between cytochrome P450 2A6 and Catechol-O-Methyltransferase genes and their association with smoking risk in young men
Source: Behav Brain Funct. 2017 May 4;13:8. doi: 10.1186/s12993-017-0127-2 (PMC5418756; doi:10.1186/s12993-017-0127-2)
Supplement: Supplementary file 3 — Additional file 3: Table S3. Comparisons of FTND, PCDS and CWS-21 among subjects carrying different CYP2A6 and COMT polymorphisms. [file 12993_2017_127_MOESM3_ESM.docx]

**Additional file 3: Table S3** Comparisons of FIND, PCDS and CWS-21 among subjects carrying different CYP2A6 and COMT polymorphisms

|  | **FTND**  **Mean (SD)** | **PCDS**  **Mean (SD)** | **CWS-21**  **Mean (SD)** |
| --- | --- | --- | --- |
| **Model 1** |  |  |  |
| **CYP2A6** |  |  |  |
| Wild type | 3.90 (2.16), N = 49 | 40.56 (13.42), N = 61 | 53.11 (15.84), N = 57 |
| High activity | 3.55 (2.16), N = 96 | 35.98 (14.38), N = 121 | 53.08 (16.50), N = 110 |
| Low activity | 3.89 (2.04), N = 36 | 35.44 (14.98), N = 45 | 50.02 (19.51), N = 43 |
| P^a^ | 0.563 | 0.087 | 0.573 |
| **Model 2** |  |  |  |
| **COMT** |  |  |  |
| COMT rs4680 |  |  |  |
| Wild type | 3.73 (2.18), N = 103 | 37.42 (14.91), N = 130 | 52.79 (18.16), N = 121 |
| Variant | 3.69 (2.09), N = 78 | 36.68 (13.61), N = 97 | 52.02 (15.24), N = 89 |
| P^b^ | 0.911 | 0.700 | 0.748 |
| COMT rs165599 |  |  |  |
| Wild type | 4.04 (2.24), N = 46 | 40.35 (15.08), N = 55 | 56.14 (17.04), N = 51 |
| Variant | 3.60 (2.09), N = 135 | 36.07 (13.99), N = 172 | 51.28 (16.8), N = 159 |
| P^b^ | 0.224 | 0.054 | 0.075 |
| **Model 3** |  |  |  |
| COMT rs4680/COMT rs165599 |  |  |  |
| Wild type/wild type | 4.25 (2.71), N = 20 | 41.83 (15.99), N = 23 | 57.50 (18.63), N = 22 |
| Wild type/variant | 3.60 (2.03), N = 83 | 36.48 (14.58), N = 107 | 51.74 (17.98), N = 99 |
| Variant/wild type | 3.88 (1.84), N = 26 | 39.28 (14.55), N = 32 | 55.10 (15.99), N = 29 |
| Variant/variant | 3.60 (2.21, N = 52) | 35.40 (13.05), N = 65 | 50.53 (14.76), N = 60 |
| P^a^ | 0.615 | 0.226 | 0.309 |
| **Model 4** |  |  |  |
| **COMT rs4680 wild type** |  |  |  |
| CYP2A6 wild type | 4.00 (2.00), N = 28 | 41.15 (13.61), N = 34 | 53.35 (16.75), N = 31 |
| CYP2A6 high activity | 3.54 (2.28), N = 52 | 36.56 (15.08), N = 66 | 54.66 (17.20), N = 62 |
| CYP2A6 low activity | 3.83 (2.21), N = 23 | 35.10 (15.67), N = 30 | 48.00 (21.29), N = 28 |
| P^a^ | 0.650 | 0.217 | 0.270 |
| **Model 5** |  |  |  |
| **COMT rs4680 variant** |  |  |  |
| CYP2A6 wild type | 3.76 (2.41), N = 21 | 39.81 (13.40), N = 27 | 52.81 (15.02), N = 26 |
| CYP2A6 high activity | 3.57 (2.04), N = 44 | 35.29 (13.61), N = 55 | 51.04 (15.48), N = 48 |
| CYP2A6 low activity | 4.00 (1.78), N = 13 | 36.13 (13.99), N = 15 | 53.80 (15.63), N = 15 |
| P^a^ | 0.798 | 0.366 | 0.793 |
| **Model 6** |  |  |  |
| **COMT rs165599 wild type** |  |  |  |
| CYP2A6 wild type | 3.55 (1.57), N = 11 | 43.27 (13.37), N =15 | 55.92 (18.52), N = 13 |
| CYP2A6 high activity | 4.15 (2.38), N = 26 | 40.89 (16.15), N =28 | 58.92 (16.02), N = 26 |
| CYP2A6 low activity | 4.33 (2.65), N = 9 | 35.42 (14.45), N = 12 | 50.33 (17.54), N = 12 |
| P^a^ | 0.694 | 0.397 | 0.359 |
| **Model 7** |  |  |  |
| **COMT rs165599 variant** |  |  |  |
| CYP2A6 wild type | 4.00 (2.31), N = 38 | 39.67 (13.46), N = 46 | 52.27 (15.10), N = 44 |
| CYP2A6 high activity | 3.33 (2.05), N = 70 | 34.51 (13.55), N = 93 | 51.27 (16.31), N = 84 |
| CYP2A6 low activity | 3.74 (1.83), N = 27 | 35.45 (15.38), N = 33 | 49.90 (20.49), N = 31 |
| P^a^ | 0.262 | 0.117 | 0.836 |

^a^ANOVA, ^b^Student’s *t* test.
